# Ancient Greek votive epigraphy: Ancient religion between individual choice and cultural trends

**DOI:** 10.1371/journal.pone.0361143

**Published:** 2026-10-15

**Authors:** Moshe Blidstein

**Affiliations:** Department of General History, University of Haifa, Haifa, Israel; Ariel University, ISRAEL

## Abstract

Ancient votive inscriptions, numbering in the tens of thousands across the Mediterranean, offer direct evidence for religious practice but have rarely been studied comprehensively across time and space due to their sheer volume. This paper presents a “distant reading” analysis of approximately 11,000 Greek dedicatory inscriptions (sixth century BCE – seventh century CE) drawn primarily from the GIST/I.PHI digital corpus. By tagging inscriptions for key features (votive/dedicatory terminology, deities, motives, gender) and analyzing their distribution chronologically and geographically while controlling for the “epigraphic habit,” this study identifies large-scale trends. The results reveal a significant relative increase in the use of explicit votive terminology (forms of *euche*/*euchomai*) over time, particularly during the Roman Imperial period and Late Antiquity. This practice exhibits strong regional variation, being markedly more prevalent in Asia Minor and the East than in mainland Greece before the fourth century CE. Furthermore, the use of votive formulae correlates strongly with specific deities (e.g., Men, Meter, Hypsistos, Hosios kai Dikaios) and negatively with others (e.g., emperors, Athena). Votive terminology also appears negatively correlated with the explicit mention of other motives (like dreams or commands) and with the use of standard consecration terms. While demonstrating the potential of computational methods for identifying broad patterns of continuity and change in religious expression, the study also highlights methodological challenges (dating uncertainty, data tagging) and underscores the persistent difficulty in definitively separating shifts in epigraphic conventions from changes in underlying religious mentalities.

## Introduction

Dedication of objects or actions to a deity was a ritual practiced in all, or almost all, ancient religions in the Mediterranean area. Indeed, some scholars consider it the core ritual of Mediterranean religion, and subsume sacrifice, prayer, and other types of dedication, under the general title of “gifts for the gods” or “offering” [1–4]. Furthermore, many dedications – of all types – were commemorated in writing by an accompanying inscription, typically placed in the sanctuary of the receiving deity. These inscriptions, numbering in the tens of thousands in various languages, form a significant body of text attesting directly to the religious practice of Mediterranean populations [5]. This vast corpus, reflecting direct interactions between worshippers and the divine in formulaic language, makes votive epigraphy an especially promising subject for large-scale quantitative analysis. A few definitions are called for to clarify the discussion. In this article I will use the term “dedication” to speak of an action of giving something to a deity; a “dedicatory inscription” to speak of an inscription which mentions or accompanies such an action; “votive” or “votive ritual/practice” as a case in which a dedication was accompanied by a vow; a “votive inscription” is defined as an inscription which explicitly mentions a vow; “consecration terms” concern only Greek terms relating explicitly to giving an object to a deity or a sanctuary.

While ancient Mediterranean dedicatory and votive practices have been investigated by many scholars in the past, several significant historical questions regarding how social, religious, temporal and geographical circumstances influenced votive practice remain unanswered, or answered only for specific cases. Specifically, there are three main questions I would like to address relating to the contexts of vowing when making dedications:

1. How did practices of vowing and of inscribing vows change in the Greek-speaking Mediterranean, from Classical Greece to Late Antiquity? Were such practices always equally popular among dedicators of objects to the gods, or were there changes in popularity, perhaps correlated with other changes in religious culture? What may explain such changes?
2. Were there significant differences between areas of the Greek-speaking Mediterranean in practices of vowing and of inscribing vows related to dedications? And if so, were these differences stable or changing over time? What may explain such differences?
3. What other circumstances may have influenced people’s propensity to vow, or inscribe their vows, when dedicating objects? Did the choice of deity to which the object was dedicated, the person’s gender, or the possible motives for dedicating, influence such practices, and why?

In order to answer these questions, this study analyzes the circumstances of Greek dedicatory inscriptions throughout antiquity, from their earliest appearance in the sixth century BCE to the seventh century CE. This is based on a digital analysis of approximately 11 thousand dedicatory inscriptions, identified within a larger corpus, forming a significant portion of all extant inscriptions of this type (some Greek inscriptions predate the sixth century BCE, but these were not included in the study due to their fragmentary condition and small number). This paper has two different audiences in mind. First, it is aimed at historians of ancient Mediterranean religion, to investigate significant trends in ancient dedicatory ritual; second, it is aimed more broadly at historians interested in a digital approach to primary sources, especially epigraphy, as an example of possible methods, their advantages and challenges.

First, I shall outline the state of the field in three areas: distant reading of Greek epigraphy, especially in religious contexts, highlighting some challenges and potentials which emerge from this literature; the historical background of the epigraphy of dedication; the understanding of the dynamics of votive practice, as it emerges from more traditional readings. Then, I shall describe the corpus, the methods of its obtainment and analysis, including data processing and limitations. After this, I will present the results of the analyses performed on the texts, and, finally, discuss their significance for the history of Mediterranean votive practices.

### The state of research

#### Historical background.

This article will focus on Greek dedicatory inscriptions in the Eastern Mediterranean, between the sixth century BCE and the seventh century CE. This long period saw many social, cultural and religious changes, which are the historical context of the creation of these inscriptions. Greek culture, including Greek religions, spread during this period over the Eastern Mediterranean and beyond due to migrations, colonization, and the political and cultural power of the Greek city-states in the Classical period, the kingdoms of the successors to Alexander the Great in the Hellenistic period, and then by the environment fostered by the Roman Empire. Sanctuaries were built throughout the region to many and varied deities with Greek names, some with long histories in the Greek-speaking sphere, others adapted from the cultures with which the Greeks interacted; these sanctuaries attracted dedications from the worshippers, many with inscriptions. In the Late Roman and early Byzantine periods, Christian ritual, belief and church formation led to new religious practices, which were, however, not a total break from the past, so that the practice of dedicating objects or even buildings to god continued despite the different ritual and institutional emphases of Christianity.

One significant religious development often noted by scholars is individuation. Though a connection between the individual and the gods (as opposed to the city or large family group) was not absent in Classical Greek religion, it became more pronounced in the Hellenistic and Roman Imperial periods [6–11]. The importance of the individual is evident in many dimensions: in the numerous possibilities open to them to shape and realize identities on both the personal and collective levels; in the growing emphasis on exceptional figures endowed with powers and with a unique connection to divine beings; and in the increasing focus on responsibility, morality, and personal destiny, as well as on the religious and moral significance of proper—and even perfect—self-formation, with close attention to the components of the self, their modes of operation, and the relationships among them. These aspects of individuation in religion became even more pronounced in Christianity. These developments may certainly have influenced also religious expression in epigraphy, including dedications [12,13]. The rate, degree and significance of change in individuation over centuries, a question much debated in scholarship, can be especially investigated through a comprehensive, long-term understanding of vowing practices in antiquity. Vows are typically performed and fulfilled by individuals; furthermore, the ritual narratives inherent in a votive inscription – the stories of the persons who had vowed to a specific deity for certain reasons, and then decided to fulfil their vows in a certain way – follow individuals in their specific interaction with deities. These individual narratives then shape the experiences of others in sacred spaces. Thus, a comprehensive investigation of votive inscriptions can shed light on when, where, and how such individuation processes impacted religious practice.

### Dedications and votive epigraphy

A dedicatory inscription would typically include a dedicatory formula or vocabulary, and either the name of the donor or the name of the deity dedicated to, or both. However, it could also be extremely short, including only one of these elements, for example, “to Athena.” On the other hand, some dedicatory inscriptions also include additional information, most notably, information on the occasion for dedication, e.g., “following a vow”, or “according to a command in a dream” [14,15]; on the motives or objectives of the dedication, for example, for the health of one’s children or parents [2,16,17]; and a description of the object dedicated, e.g., an altar or lamp.

The different possible formulae have been noted by scholars, especially the longer and more complex ones which indicate why worshippers dedicated. These have been rightly understood as providing a glimpse into the religious mentality of ancient people. However, while the relative frequency of these formulae in general has been noted, trends in the data have never been analyzed more rigorously on a large scale to date. Thus, it is unclear how representative – or not – are specific dedicatory formulae across time and space.

One outstanding question concerns the relationship between dedicatory and votive practice. Some dedications mention simply the fact of giving or consecration of the object: for example, the inscription θρα̣συκλείδες ἀνέθεκε̣ (“Thrasukleides consecrated this” – IG I^3^ 556; PHI no. 587, found in Athens and dated to the early fifth century BCE). Others, which I term votive dedications, mention that the dedication was performed following an *euche* (vow/prayer); for example, γάιος μέμμιος θρέπτος λαμπτρεὺς διὶ ὑψίστῳ εὐχήν (“Gaius Memmius Threptos, from Lamptrai, [dedicated] a vow to Zeus Hypsistos” – IG II^2^ 4737, from the first or second century CE). Though *euche* may mean either vow or prayer, when this term is found in an inscription connected to a dedicated object it is commonly assumed to refer to the vow that preceded the dedication rather than a prayer (though this is more debated in Christian contexts [18]). If this statement indeed reflects actual practice rather than a formula only, it shows that the dedication was the culmination of a long process of negotiation between the worshipper and the deity. A vow could be the result of a difficulty in life, or the expectation that a difficulty may arise, for example, in case of illness, before a long journey, an athletic contest, or economic woes [1,4,17,19–23]. However, it could also be an action performed from time to time without a special reason, to assure the goodwill of the gods, and as part of a general request for health and prosperity, made periodically, or as a sign of joy and thankfulness for benefits given. A vow could commonly be performed for the benefit of others, whether family members, honorands or rulers. Again, only rarely does the text include information on the vow itself, which would allow us to understand its timeframe, motives and objectives. Of course, vows may have accompanied dedications also without being mentioned in the inscription, but there is no reason to assume they were performed in this case, since a dedication can be made without a vow. Thus, I emphasize that this study is based solely on the terminology of the inscription, which may be a result of many other considerations beyond reflecting which rituals actually took place at the time of dedication, such as copying common formulae.

Of course, the objects dedicated, the inscriptions accompanying them, as well as literary depictions of dedicatory practice were the subjects of many studies in the past [1,14,16,19–21,24–36]. Nevertheless, the research questions stated in the introduction regarding chronological, geographical and thematic developments in votive practice have never been studied on the epigraphic corpus as a whole, or even on a large section of it. Considering the large number of texts involved, studies in the past have tended to focus only on a small part of the corpus: typically, on specific areas, periods, deities, or aspects. Angelos Chaniotis has argued, “Studies of epigraphic habits are comparative studies, requiring comparisons between sites, regions, periods, types of documents, and different social groups” [37]. Past studies have shown why and how people vowed according to specific inscriptions, but the lack of a wide comparative framework situating these inscriptions in the context of more general trends of votive practice reduces their explanatory power. Thus, while we know that people vowed in certain occasions, we do not yet know how typical this was of their time and place, or what other factors usually influenced their choice to make a vow. Comparative studies of epigraphy add significant dimensions to our knowledge, but they are difficult for many technical reasons. The development of digital platforms which include a large part of the Greek epigraphic corpus, and thus the availability of the texts for digital or “distant” reading, changes this situation.

Since most inscriptions have a clear geographical provenance, and many of them have been dated at least to a general time span, this data can be used to investigate not only when vowing became more frequent, but also the geographical contours of such a shift, i.e., whether vowing rather than simply dedicating characterized specific places or areas in certain times. Moreover, using computational analysis of this large dataset, it is possible to investigate the relationship between vowing and other data appearing in the inscriptions: did people inscribe their vows more when they dedicated to certain deities, or when they had specific motives? How did the ritual of vowing and inscribing vows develop over time and over space?

I have been inspired in this regard by the work of Ulrich Ehmig, especially her book *Donum Dedit* [38], which analyses the corpus of Latin dedication inscriptions, especially focusing on the comparison of those mentioning vows (*votum*) to those which are gifts without vows (*donum*). Ehmig’s most important insight is that the choice whether to give a votive or a gift to the gods was not random, but was a conscious and calculated decision on the part of the giver, which can be correlated with other geographical, temporal, cultural and social variables [38]. In this I follow also the suggestion of Rudolf Haensch [34], who comments that such work is needed also in the Greek sphere. Indeed, there is no similar analysis of Greek dedication inscriptions. Such a systematic comparison between dedication inscriptions with and without votive terms and their connection to different ritual scenarios and social situations, can help correlate votive behaviour with chronological and geographical shifts in religious mentalities and institutions, and thus to understand their meaning for the population.

Some scholars have argued that the epigraphic data reflects a general shift in religiosity during the first centuries CE, in which people expressed more intimate relationships with their deities [20,39]. However, these arguments (and others like them) are always generalized from a small number of inscriptions, without any attempt at comprehensiveness. A careful investigation of the votive character of a large number of inscriptions can provide robust support or refutation for this argument.

Furthermore, it is clear that significant changes occurred also in the late fourth to sixth centuries, presumably as a result of broad Christianization of the population. Though Christians, like adherents to traditional Greek religions, continued to vow and record their vows in inscriptions, they used slightly different formulas, vowed objects to churches rather than temples, and invoked Christian deities rather than pagan ones [34,40–50]. However, without a comprehensive analysis of all dedication inscriptions until now, it is unclear whether other dimensions of votive practice changed as well, for example, whether the motives for vowing also remained stable with Christianization.

Since the dataset does not include metadata on the character of the findspot (e.g., church, temple or synagogue), we can only be guided here by the language of the inscription itself. It is important to note some methodological problems with the very notion of Christianization and the identification of inscriptions as Christian or otherwise. First, there may be at times a circular logic at play, since non-dated inscriptions with Christian features were sometimes dated by editors to after the late fourth century simply because of these features (and, to some extent, vice versa: dedications to pagan gods were dated earlier). Such dating conventions may mask a more significant mixing of Christian and pagan in the fourth and fifth centuries, or even beyond. Second, many people may not have clearly identified as either Christian or pagan in their religious practice, or even if they did, they may have used formulae more typical of the “other” group on some occasions, as in well-known from accusations in the church fathers of “pagan” behavior by church-goers. Such cases would be missed if we simply rely on dating and on epigraphic formulae to separate Christian from pagan for which closer readings of the inscriptions in their material and spatial contexts are needed. In light of these issues, I refrain from trying to define some votive or dedicatory practices as Christian or otherwise, and rather chart the internal characteristics of the formulae over time and space.

An important issue which will not by developed in detail here concerns differences between votive practices in different languages. It has been argued, for example, that the different formulas used in Latin vs. Palmyrene Aramaic attest to a radically different religious mentality, not only to a different epigraphic habit (e.g.[51]). However, such issues remain to be investigated in the future through comparative cross-linguistic studies.

It is important to note that in many areas Greek was not the only language in which religious ritual was practiced and inscriptions written, so that the Greek inscriptions form a subset of all inscriptions from the region, in Greek, Latin, Aramaic, Semitic (e.g., Hebrew, Aramaic, Nabatean), Egyptian, and Anatolian (e.g., Lydian, Lycian) languages. Thus, the choice to study Greek inscriptions alone, which is a result of the technical availability of the data and the limitations of one study, may skew the results towards the practices of Greek-speaking inhabitants of the region. Furthermore, since many people in the region were bi- or even trilingual, the selection of Greek may produce an artificial focus on one part of a ritual whole.

### Distant reading of Greek epigraphy

Computerized analysis of epigraphic material has been applied especially to Latin epigraphy, where much work has been done towards making the texts and metadata comprehensively and freely available to the scholarly community in online corpora. These were mostly used for the facilitation of traditional close readings, but some distant readings, performing statistical analysis on the whole corpus or on large parts of it, have also been published. Furthermore, machine learning techniques have been applied to epigraphic corpora, for example to assist in restoring lacunae and fragmentary texts, or in classifying them into different types. These indicate many potential pathways for how such corpora can be utilized for social and cultural history [52]. Several important studies have outlined best practices and possible applications for such analysis, especially concerning geographical patterns, including methods such as spatial statistics or network visualization.

The digitization of Greek literature has been underway for several decades, so that computerized analysis of literature using distant reading methods, though hardly mainstream, has already been possible for a while (e.g., corpus construction, studies on authorship attribution, text reuse or semantic analysis [53]). Greek epigraphy has been a bit late to this trend. This is possibly a result of the unique features of these texts, both linguistically and historically speaking. For example, the orthography of epigraphy is much more diverse and localized. Furthermore, factors like extensive fragmentation, the prevalence of abbreviations, and significant dialectal variations pose considerable challenges for automated processing. Nevertheless, in the past few years this area has developed and a few important trends should be noted. First is simply the fact that a large number of inscriptions have been made publicly available in several stages, and crucially, enriched with extensive metadata [54,55]. Second, work has been advancing on the development of lemmatization techniques for Greek epigraphy, since lemmatizers trained on literary texts produce very poor results when applied to epigraphic texts [56]. Lemmatization is essential for semantic analysis as it groups together words from the same root. Building on these resources, it has been possible to examine several questions on religious history in the Greek Mediterranean, as can be seen in the work of Tomas Glomb, Vojtěc Kaše, Adela Sobotková and Petra Heřmánková [57].

One avenue of research is geographical: using the chronological and geographical metadata of the inscriptions in order to locate and analyze patterns of diffusion and movement of practices and ideas, and to examine links between developing phenomena reflected in the epigraphy and other variables, for example, the presence of military, political or professional personnel and institutions who may have had a role in spreading such ideas and practices [58,59]. This avenue is strongly connected to the idea of applying network analysis to understand the connection between the transportation network (by land and sea) or networks of institutions, such as churches, and the diffusion of ideas and practices [60–62]. Focusing on the chronological dimension is also possible, in order to evaluate possible connections between significant events (e.g., plagues) or trends (religious affluence) and the rise of cultural phenomena [63,64]. Epigraphic texts had been recognized as especially relevant for such an analysis because of their robust geographical placing relative to literary texts; however, their dating is often very imprecise, requiring either using only inscriptions with robust dating, significantly reducing the amount of data, or the use of various methods to model temporal uncertainty [65].

However, as Bodel, Prag, and Roueché [66] argue, the application of these computational methods requires a critical re-evaluation of the data sources themselves. The creation of digital corpora reinforces their perception and treatment as neutral aggregations of data, obscuring the specific editorial choices, authorship, and the material and historical context of the texts being analyzed. The usual practice of citing inscriptions by abstract identifiers (whether traditional corpus numbers or digital IDs) creates a situation in which distinct editions created by different scholars on different bases are treated as identical data points. While this result can be celebrated as facilitating new perspectives on the data [67], it also presents a significant risk: results may reflect the coverage biases and editorial conventions of the underlying datasets rather than genuine historical patterns. Consequently, valid computational analysis depends not only on the availability of text, but on an understanding that the corpora rests on several layers of scholarly interpretation and bias. A prime example of this bias is geographic representation: dedications and inscriptions are naturally found in greater numbers in sites and areas which have been subject to more intense archaeological study, due to factors such as greater public interest and funding or political stability and security challenges. Furthermore, continued inhabitance at many urban sites prevent comprehensive archaeological investigation, or led to the dismantling of ancient sites and the dispersal of their inscriptions in the intervening periods [68].

## Materials and methods

### Corpus definition

#### Base corpus: characteristics and limitations.

The base corpus used for analysis is the GIST dataset (v1.1 [55]), based on the I.PHI dataset [54]. GIST consists of 217,863 Greek inscriptions, including cleaned and lemmatized texts, and, crucially for this study, extensive chronological and geographical metadata. I.PHI was formed for purposes of research on reconstruction of lacunae in Greek Epigraphy using machine learning; as such, its aim was to present a unified and clean textual corpus, clearly structured by place and time, and was not interested in other aspects of inscriptions such as archaeological context, supporting material or accompanying artwork. GIST inherited I.PHI, with further deduplication, cleaning and updated lemmatization, and added mostly geographic metadata. Both datasets use texts which are basically those of the largest online site for Greek epigraphy, the Packard Humanities Institute (PHI) [69], and much of the metadata is also derived from data found on that site. The PHI text and metadata, in turn, is derived from major print editions, especially but not limited to the *Inscriptiones Graecae* project, some from the beginning of the 20^th^ century.

As an example, inscription with ID 240365 is the following: “ἀπόλλωνι πυθίωι εὐξάμενος ἀνέθηκε χαιρέφιλος φείδωνος ἀθηναῖος” (Having made a vow, Chairephilos son of Pheidon of Athens dedicated (this) to Apollo Pythios). The inscription was published in traditional corpora (Fouilles de Delphes III 4.204, SIG3 201) and appeared in the PHI site with the metadata, “Phokis — Delphi — stoich. — 350-300 BC — SIG(3) 301”. From this data, and by cross reference to other datasets, I.PHI and GIST produced geographical and chronological fields (Longitude 22.5014055, Latitude 38.4819545; not_before −350, not_after −300). I note that this inscription also appears in Attic Inscriptions Online [70] with a more precise dating of 332/1 BCE according to more recent publications, but such information does not appear in the datasets and therefore could not be included in this study.

All analysis was performed using Python (v3.12) and the Pandas library (v. 2.2).

The characteristics of the corpus are very significant for the following analysis. On the positive side, this is a very large corpus. It is rare that historians of antiquity can muster such a large number of texts for studying a subject, and to the best of my knowledge the largest Greek epigraphic dataset digitally available. The inclusion of findspots in the corpus metadata for most of the inscriptions is highly significant for spatial analysis.

However, there are also some significant problems or challenges with the corpus:

1. Coverage: While this is a very large corpus, it does not provide comprehensive coverage of all known Greek inscriptions, because not all known publications were included in the original I.PHI dataset. Specifically, publications of the last decade or so were generally not included.
2. Representativity: Such epigraphic corpora are by no means representative of the ancient population, or even of ancient epigraphical activity. This is certainly true of our corpus as well.
3. Dating: A significant challenge is the chronological information. Many inscriptions in the corpus lack dates altogether; others are dated to a range, often of a century or more. This study attempts to mitigate this problem in several ways First, inscriptions lacking chronological metadata were excluded entirely. Second, where date ranges were provided, inscriptions were modeled probabilistically across their full attested range rather than assigned to a single point in time, as this creates artificial peaks in the midpoint date of common ranges. Instead, for analyses requiring temporal distribution (Fig 1, 3-9), each inscription with a date range was represented probabilistically: it was duplicated 10 times, with each instance assigned a date uniformly distributed across its possible range (e.g., an inscription dated 100–190 CE was duplicated to instances dated 100, 110,... 190 CE). For the proportional analyses mostly used in this paper, this models the uncertainty without introducing artificial peaks. For absolute counts, results were divided by 10. I considered alternative approaches like Bayesian modeling but opted for this method due to its simplicity and suitability for calculating relative proportions (see [65] for possible methods). This was seen as suitable also considering the temporal range of this study, which is broad and does not attempt to find temporal patterns of less than a century. Third, the analysis consistently uses relative proportions (e.g., votive dedications as a percentage of all dedications) rather than absolute counts, reducing sensitivity to uneven preservation and to the “epigraphic habit.” Fourth, I generally use 100-year bins in the analysis (for discussion of the appropriate size of dating bins in the study of epigraphic material, see [71]). Since a large proportion of the inscriptions are dated only broadly, finer chronological divisions would create an artificial precision and obscure rather than clarify long-term trends. The use of century-level bins therefore reflects the chronological resolution of the material and allows for the identification of changes that operate over extended periods.

**Fig 1:**
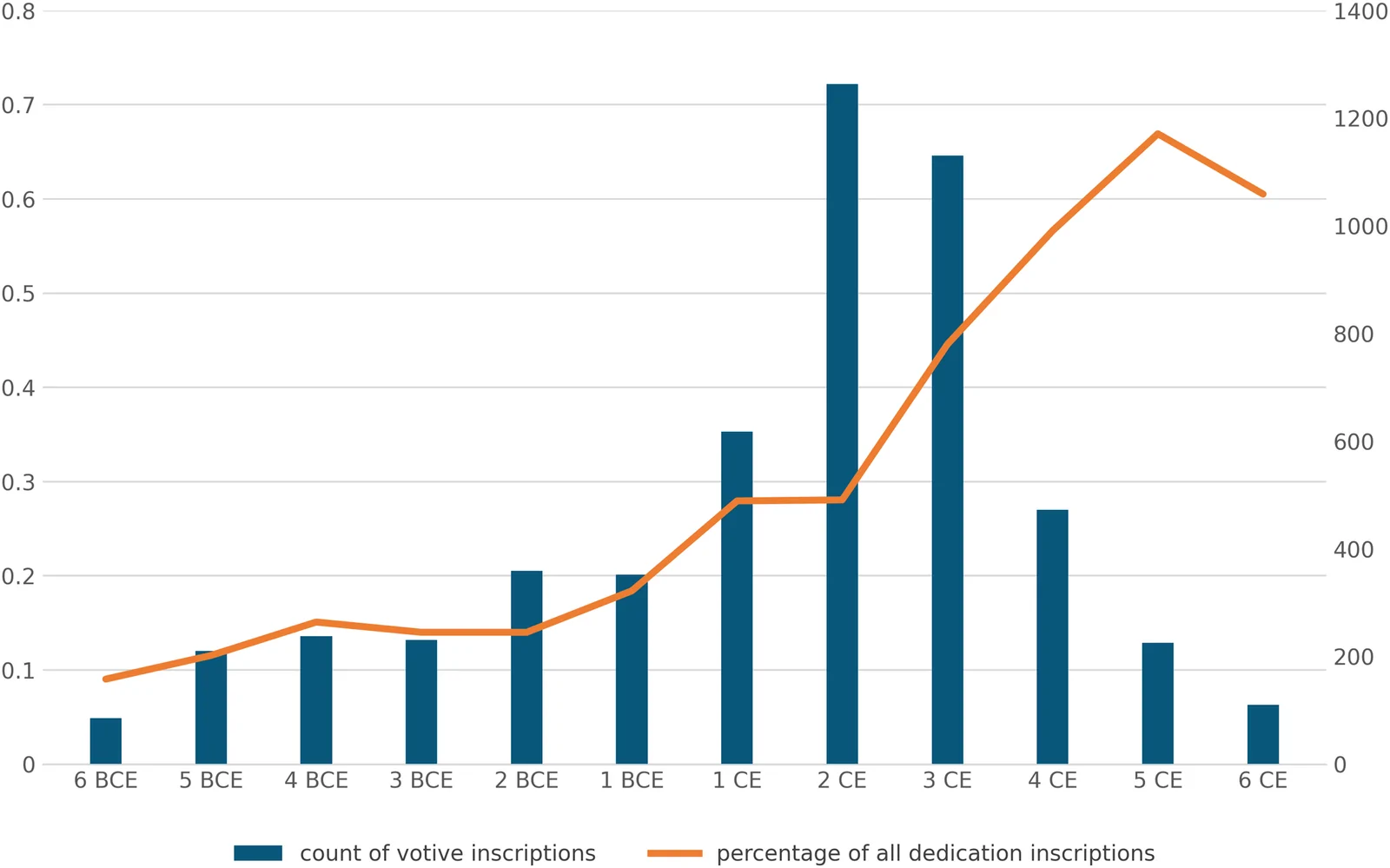
Votive inscriptions over time. Votive inscriptions, count and proportion of all dedications.

1. Material: The dataset does not include data on the material inscribed or on any other material aspect. Therefore, texts inscribed or written on stone, mosaic, metal or pottery are all included, with no way to differentiate between them; there is no differentiation between, e.g., monumental lettering or graffiti, despite the clear difference such aspects would have had for worshippers, writers and readers of these texts.
2. Fragmentary and lacunose texts: Epigraphic texts are very frequently difficult to read, composed of several fragments, or missing large portions. The GIST dataset includes fields for several options of representing the text of each inscription, inherited from the I.PHI corpus and study [72]: the text as published, with editorial marks and special symbols (the field “text_raw”); the text with special marks and symbols removed, with missing text represented by brackets (“text_iphi”); the text without any restorations or symbols (“clean_text_conservative”); and a text with restorations and expansions by editors (“clean_text_interpretative_word”). I opted for using the latter, restored text. On the one hand, this certainly introduces some doubtful restorations; on the other hand, it allows using highly probable restorations. Since dedication and votive inscriptions are formulaic, it is certainly possible that certain editors would restore according to common conventions, e.g., adding “*kat’ euche*” to a broken off dedication inscription, thus artificially skewing the results deriving from specific corpora. Thus, I will check to what degree restorations insert relevant information.

Geographically, I used the largest division in the metadata into 12 regions. This leads to a problem of very large areas of comparison, some of which are very heterogenous; for example, an inscription from the Armenian area and one from Constantinopolis will be put together under one region despite the huge cultural and geographic distance between them. The borders between these regions are to some extent arbitrary, and may reflect modern understandings of ancient geography. The corpus metadata also includes sub-regions. However, using smaller sub-divisions would result in much smaller numbers of dedication inscriptions in each, which would make comparison very difficult, especially if attempting to divide further by time or other factors. The high level of aggregation allows the identification of contrasts between areas that repeatedly emerge across centuries and across multiple analytical dimensions (terminology, deities, motives). In several cases, the results point to the need for more fine-grained, contextually grounded follow-up studies at the level of individual sanctuaries or sub-regions. In this sense, the regional analysis useful for indicating where closer investigation is most likely to be productive, rather than for providing definitive geographical significance of the data. Despite the problems, geographical division is worthwhile and provides more insight than simply looking at all the inscriptions as a whole without any differentiation into regions. Future studies may attempt to divide the corpus into regions or clusters based on data from the inscriptions themselves, for example by analyzing differences in language.

#### Sub corpus construction.

Since my main objective was to understand why and when people decided to dedicate objects with a vow, the primary step was to select a sub-corpus of dedicatory inscriptions. However, the GIST metadata understandably does not include the type or genre of inscription (e.g., funerary, honorary, dedication etc.), since this is a subjective categorization, invented by scholars not ancients, and is not always clearly marked lexically [20]. For example, inscriptions on churches stating their contributor are commonly categorized as ‘building inscriptions’, though they differ little from dedications [46]; similarly, the line between funerary inscriptions dedicated to the soul of the dead and votive inscriptions to divinities can be blurred [50,73]. Furthermore, honorary inscriptions to divinized rulers and other humans can sometimes be very similar to, or form complex interactions with, dedications to divinities [74–76]. These problems do not prevent the discipline from using such categorization, however, as all major corpora (e.g., the SEG) use some form of it [77,78]. It was decided to include all “building inscriptions” in which a building (usually a church or temple) was dedicated to a deity, as well as inscriptions dedicating objects to the Roman Emperor or his temples; epitaphs dedicated to the spirits of the dead were not included.

Therefore, what was needed was an iterative process of identifying how dedication inscriptions are defined in existing scholarship and extrapolating to inscriptions not yet identified as such. Thus, a multi-step pipeline was implemented:

1. Identifying potential dedications by collating publication identifiers from existing corpora/lists known to contain relevant genre metadata:
  a) Inscriptiones Graecae [79] (“weihinschrift” and “votivinschrift”, 1604 inscriptions)
  b) Inscriptions of Israel/Palestine [80] (“dedicatory” of all types; 466 inscriptions)
  c) Mapping Ancient Polytheisms [81] (“building dedication, dedication”; 5781 inscriptions)
  d) Associations in the Greco-Roman World [82] (Building dedication or inscription; Dedication inscription; 504 inscriptions)
  e) Attic Inscriptions Online – AIO [70] (Dedication (private), Dedication (public); 1259 inscriptions)
  f) Inscriptiones Christianae Graecae – ICG [83] (Building dedication, dedication; 171 inscriptions)

All of these categorize inscriptions by genre with a category for dedications, thus creating a significant list of dedication inscriptions already identified as such by the scholarly community, which could be used as a ground truth (GT). Many of these lists overlapped. The SEG [84] and Kernos Epigraphic Bulletin [85] were also consulted for categorization but their publication identifiers were not used.

2. GIST includes a field for publication identifiers – i.e., publication details, IG volume and number, or SEG volume and number. Since the GT also commonly include these identifiers, it was possible to identify the majority of the inscriptions from the GT in the GIST corpus. Furthermore, the GT was examined to produce criteria to identify additional inscriptions as dedications: votive terms, consecration terms, mentions of deities, and inscription length. These criteria were not fully equivalent. The presence of votive terms (*euche*/*euchomai*) or motive terms always implied a dedication. The presence of consecration terms (e.g., *anatithemi*) as well as the presence of deity names in the dative case usually implied dedications but these were also present in some epitaphs. It was also found that the vast majority of dedications inscriptions in the GT were short.

3. In the next stage, these criteria were used to populate a list of additional potential dedications, with
  a. inscriptions containing votive or motive terms, no matter their length (a list of terms is in S1 table);
  b. inscriptions under 200 characters with a deity in the dative and a consecration term (a list of deities and terms in S1 table).
  c. inscriptions under 50 characters and a deity in the dative only, to include short dedication inscriptions mentioning only the deity’s name, or only the deity and dedicator.

These criteria were checked against the GT.

4. Inscriptions without geographic and chronological metadata in GIST were removed from this list.
5. This list was then examined and was found to contain mostly dedications but also some epitaphs (with consecration terms or curses containing deity names). These were programmatically excluded based on keywords (e.g., removing any inscription containing terms for “tomb” or “buried” like *taphos*, *mnema*, etc.).
6. Finally, 20% of the resulting sub-corpus was randomly sampled to verify that it consists overwhelmingly of dedications.

While some unusual dedications may have been missed in this procedure, it is based both on the cumulative expertise of major existing editions (the IG), on the major tools of the field (SEG), and experts and projects in epigraphy relating to Greek, Jewish and Christian religion (Kernos Epigraphic Bulletin, Inscriptions of Israel/Palestine, ICG, Mapping Ancient Polytheisms), as well as on manual verification. This procedure yielded a sub-corpus of 10,795 dedicatory inscriptions with associated chronological and geographic metadata from GIST.

### Corpus analysis: tagging and quantification

The next step was to tag the corpus of dedicatory inscriptions to facilitate digital analysis. For each inscription, I attempted to find the following information (the full list of tags is in S1 Table):

1. Divinities – Names of divinities and epithets mentioned in the dedication (87 tags)
2. Reasons – on behalf of whom the dedication was given: e.g., on behalf of the emperor or family members, for well-being or salvation (soteria) (21 tags)
3. Motives – why the dedication was given (e.g., as first-fruits, tithe, following a dream, or following a commandment) (6 tags)
4. Consecration terms *– anatithemi, dora, anathema, kathiero* (4 tags)
5. Votive terms – *euche*, *euchomai* (2 tags)
6. Gender of the dedicator (2 tags)

These specific tags were chosen because they directly address key questions about the agents (gender), recipients (divinities), context (reasons, motives), and terminology (consecration, votive) of the practice. Tags were structured by a triple level ontology: category-item-detail, e.g., “gods – Athena – Lindia” or “reasons – family - daughter” to allow for both general and detailed analysis if needed, each level on a separate column, though most tags included only a category and item. For the purposes of this study, I used only the category and item levels.

To return to our example, inscription ID 240365, was tagged with the following tags: “dedication terms - anatithemi”, “gods – apollo”, “votive terms – euche”.

Tagging produces metadata by grouping significant lexemes under a categorical group. This metadata can then be analyzed for inter-correlations and chronological or geographical trends. While tagging is essential in order not to discuss each lexeme separately, it also has the potential for skewing the results if the tagging procedure locates only part of the relevant lexemes or locates non-relevant lexemes. The tagging was done by identifying a group of regex matches related to each tag, and tagging inscriptions including these regexes (see S1 Table for a list). For most cases, single lexemes were used, but for motives and reasons I used wildcards to capture expressions more flexibly.

I matched the regexes to the text itself rather than stemmed or lemmatized forms of the lexemes, as in some cases where declension or conjugation made a difference, for example whether the emperor appeared as a deity to which the dedication is given (in the dative) or as the one on behalf of whom it is given (generally in the genitive and preceded by *hyper*); also, using lemmatized text matched many superfluous texts because of irrelevant homonyms and incorrect lemmatization. The identification of the required regexes was an iterative process, in which I manually checked 20% of the tagged inscriptions to see whether they were tagged correctly and comprehensively. For deity names, I used the database of the MAP project [81], a comprehensive database of deity names and epithets in different forms; for terms relating to reasons, motives, consecration and vowing, I consulted relevant publications [2,3,16,17,26,86].

An important point concerning the deity tags is that they are individual; that is, an inscriptions mentioning the deity “Zeus Dionysus” is parsed as two separate tags, Zeus and Dionysus, and so also epithets, e.g., “Aphrodite Ourania” (Heavenly Aphrodite). The reasoning for this is that the relationship between parts of divine names are famously complex [87,88], so it is impossible in the confines of this study to disentangle them or to decide whether, e.g., Zeus Soter is the same or different deity from Zeus Bronton. Furthermore, treating each and every name version as separate would lead to a huge multiplication of deities and a much smaller number of inscriptions for each, and make comparisons very difficult.

The gender tag required additional information in order to determine the gender. Here, I used the online Lexicon of Greek Personal Names (LGPN) database [89], which includes the gender of each attested name, to decide whether a dedicator was male or female. In cases of ambiguity – names which were commonly attested for both genders or inscriptions with many names – I did not tag for gender. Additionally, for each inscription I retained the chronological and geographic information given in GIST. These tags, together with the GIST metadata, were the basis for the analytics I performed on the inscriptions. Thus, in the case of the example ID 240365, I could tag the dedicator as a male because his name appears as such in the LGPN.

All the tags keyed to PHI number were published as an open dataset in Zenodo [90].

A core challenge in diachronic analysis of inscriptions is the extreme heterogeneity of the epigraphic practice, known as the epigraphic habit. In brief, the custom of inscribing texts on stone in certain periods was much more prevalent than in others, with distinct peaks in the Hellenistic and High Roman periods, though there is much diversity in this habit also between different places [91,92]. Moreover, the absolute number of inscriptions is also a result of vagaries of preservation and discovery. Therefore, simple counting of votive or dedicatory inscriptions cannot be an index for changes in votive practice. Thus, for this analysis I mostly used the number of votive inscriptions (i.e., those tagged with “votive terms”) as a **percentage** of all dedication inscriptions. There is no a priori reason to assume dedication inscriptions containing votive terms were differentially preserved or discovered compared to other dedications, or that an overall rise in people’s propensity to add inscriptions to their dedications would be accompanied by a rise in votive terms as a percentage of these inscriptions. When such changes over time and space are found, it would therefore be reasonable to conclude that they were a result of an actual change in religious practices and attitudes. To better understand such changes, I then examined the differences in this percentage for inscriptions marked by various dimensions (consecration terms, deities, motives, reasons, time and space).

Specifically, to determine which specific deities were significantly associated with the use of votive terminology after accounting for temporal and regional variations, I conducted a statistical analysis using logistic regression. In this case, the dataset was aggregated to the inscription level (N = 10,795), with the primary outcome being a binary variable indicating the presence (1) or absence (0) of a votive term (has_votive_term). Inscriptions lacking century data were excluded. For each deity, a separate logistic regression model was fitted using statsmodels in Python, predicting has_votive_term based on the presence of that specific deity (has_god), while controlling for categorical representations of inscription century (C(century_cat)) and region (C(region)). The significance threshold (alpha) was set at 0.05. I recognize that testing each deity individually involves multiple comparisons. However, no formal correction for multiple comparisons was applied due to the exploratory nature of identifying individual deity associations; results were interpreted accordingly. Models were checked for predictor variation and issues like non-convergence or perfect separation. For this specific regression analysis, the date duplication method was not used, as it would interfere with the significance test. Instead, the midpoint date of the inscription’s range was used for assigning century in the model. Considering the broad range of the period investigated (1400 years) this appeared legitimate.

Restorations could skew the results. I could not examine this comprehensively for all subjects, but focused on votive terminology and its distribution, as this is the core of the article. I first checked how many inscriptions tagged as containing votive terms in the restored text did not contain such terms in the non-restored, “conservative” text: the number was quite high, 536 out of 2201 (24%). However, an examination of the non-restored text in these cases shows that 406 of these inscriptions contain some letters of the putative votive term, e.g., “*euch*”, making the restoration highly probable, while only 130 (6% of all inscriptions with votive terms in the restored text) were heavy restorations in which there is no trace of the votive term in the conservative text. As I would not want to discard these 130 inscriptions completely, it is worthwhile to understand their chronological and geographical distribution compared to the rest of the votive inscription corpus. They are much more prevalent in earlier periods – 23% of Archaic and 15% of Classical inscriptions in the votive corpus contain heavily restored votive terms, compared to only 4%−6% in later periods. Presumably, this is a result of the more fragmentary condition of earlier inscriptions. Thus, if some of these restorations are false, this means that vows are somewhat less attested in earlier periods than would appear at first.Geographically, of areas with more than five heavily restored inscriptions with votive terms, these are more prevalent in Attica (14%), Thrace and the Aegean (8% each), and less so in Cyprus and North Greece (6%) or Asia Minor (4%). A reviewer suggested that because publication of inscriptions from different areas were performed over the past century, some areas were published early in the twentieth century, during a period in which expansive restorations were more accepted by scholars.

## Results

### Votive dedications over time and space

Overall, out of 10,795 Greek dedication inscriptions in the corpus, 2201 (20%) contain votive terms. However, the distribution of such terms in both time and space is highly heterogenous.

Fig 1 shows there is a clear trend in which votive terminology becomes much more prevalent, both numerically and relative to the number of the overall dedications. Thus in early Classical Greece (sixth century BCE) words of the root *euche*/ *euchomai* appear only in 9% of the inscriptions, in the Classical and Hellenistic period (fifth-second century BCE) this rises to 13–18%. In the imperial Roman period there is a jump to about 28%, and a further rise to 44% in the third century CE. In the fourth century there is a significant fall in the absolute number of inscriptions but the relative number of votive inscriptions becomes even higher – 57%, rising to above 60% in the fifth-sixth centuries CE. This trend indicates a clear, if slow and nonuniform, expansion of votive terminology in the Greek world. The expansion is even more marked when reminded of the high number of restored votive terms in the Archaic and Classical inscriptions. The rising prevalence of votive dedications shows that false restorations are probably not at play, since, as shown above, restorations are significantly more prevalent in earlier inscriptions.

The expansion should also be analyzed geographically. Since the use of Greek in speech and inscriptions expanded geographically over time, from the area of modern Greece and west Asia Minor to a much larger area, the rise in votive terminology may be a matter not of change in the Greek lands of origin but a process in which places in which vows were already customary started to express this in (Greek) inscriptions. This question can be empirically examined by checking the geographic contours of the expansion. The evidence shows that in some areas vowing was customary, and generally on a rise through time, while in others it was quite rare (Fig 2). This phenomenon should be linked to the general “epigraphic curves” of the different Roman provinces [91,93], where we find a first peak in the Hellenistic period, and then a more distinct peak in the Imperial Roman period. In the votive inscriptions, however, the Hellenistic peak is hardly found.

**Fig 2:**
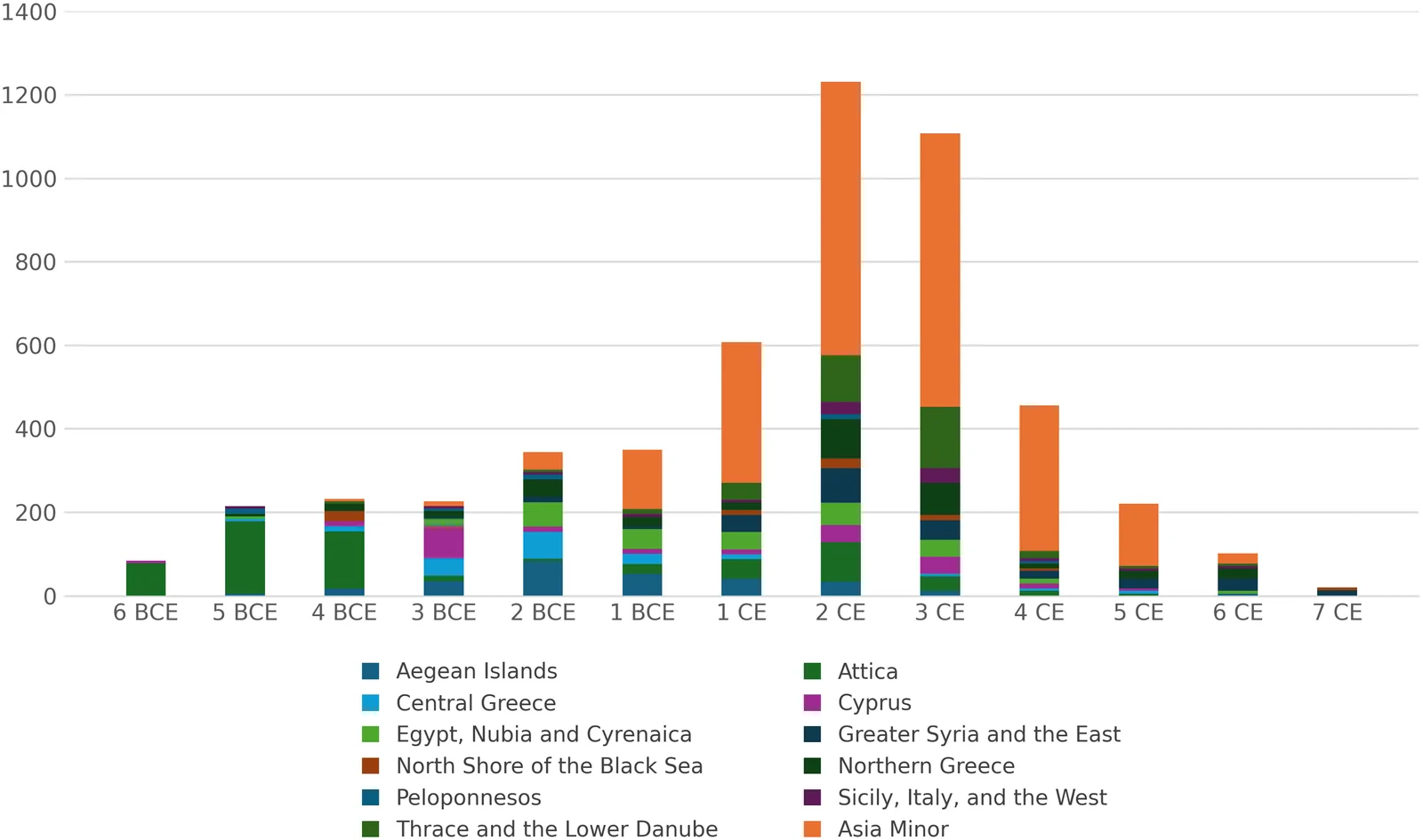
Regional count of votive inscriptions.

As can be seen from Fig 2, in the Roman period the number of votive inscriptions in Asia Minor greatly exceeds other provinces, accounting to more than half of the inscriptions, and this preponderance becomes even more significant in the fourth and fifth centuries. The importance of vows in ecclesiastical building inscriptions from Asia Minor relative to Syria and Italy was noted by Rudolf Haensch [46].

However, due to the epigraphic habit, relative proportions are more informative for comparing regional practices. The general distribution of votive vs. non-votive dedications can be seen in Fig 3, a map focusing on the Eastern Mediterranean and comparing the two types of dedications. Asia Minor, Northern Greece, Thrace and the Lower Danube areas have many votive dedications, while Attica, southern Greece, the Aegean, as well as areas of Greek colonization – the Black sea, Sicily, and Alexandria – have large amounts of non-votive dedications.

**Fig 3:**
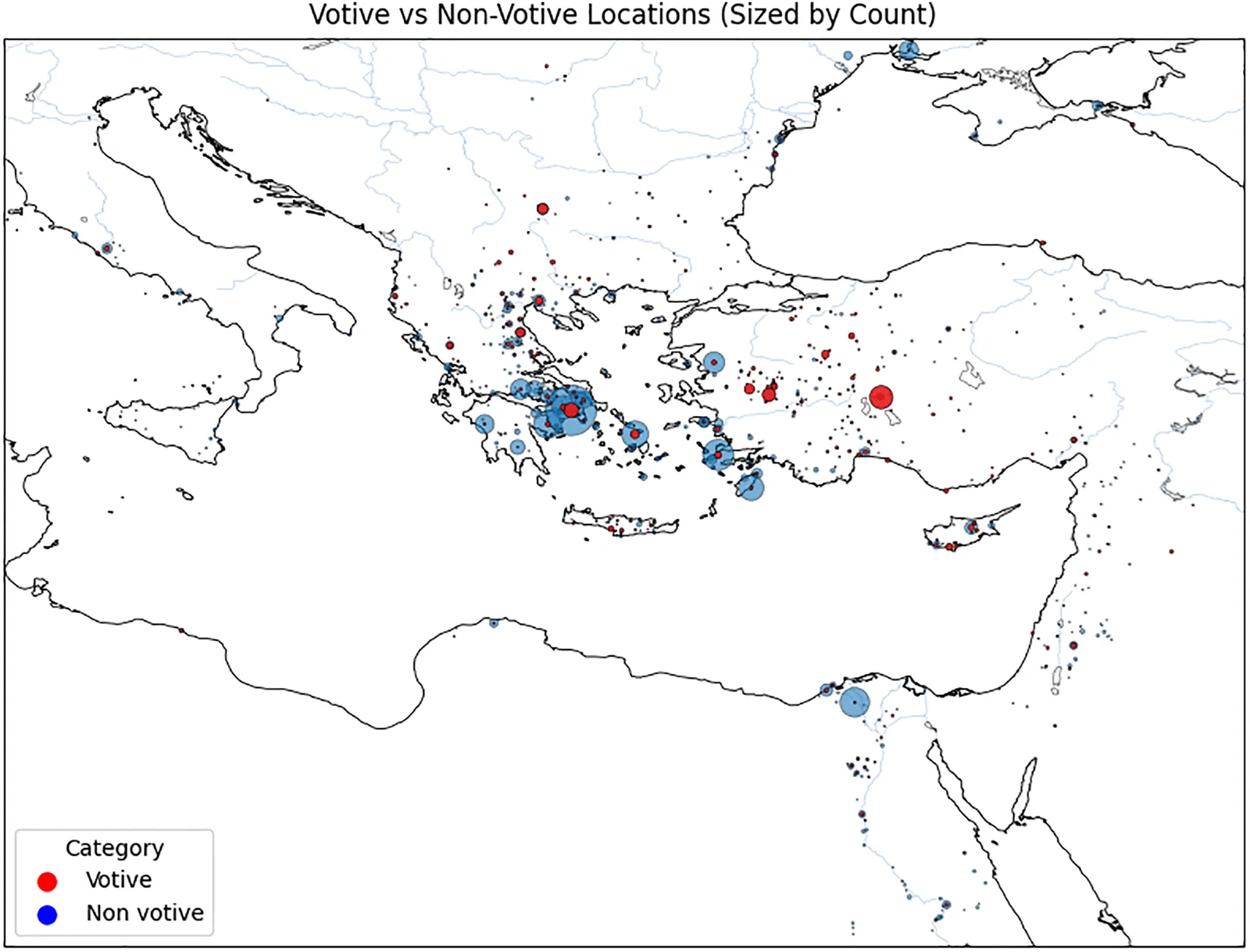
Dedications in the Mediterranean. The Mediterranean with votive and non-votive Greek dedication inscriptions. Map created in Python using Cartopy with coastline, river, and lake vector data from the Natural Earth dataset (public domain).

The map in Fig 3 provides a general visualization, but more rigorous information with a temporal breakdown can be seen in Fig 4 by plotting these proportions (votive dedications as % of all dedications) over time for 12 major regions (excluding two with minimal data).

**Fig 4:**
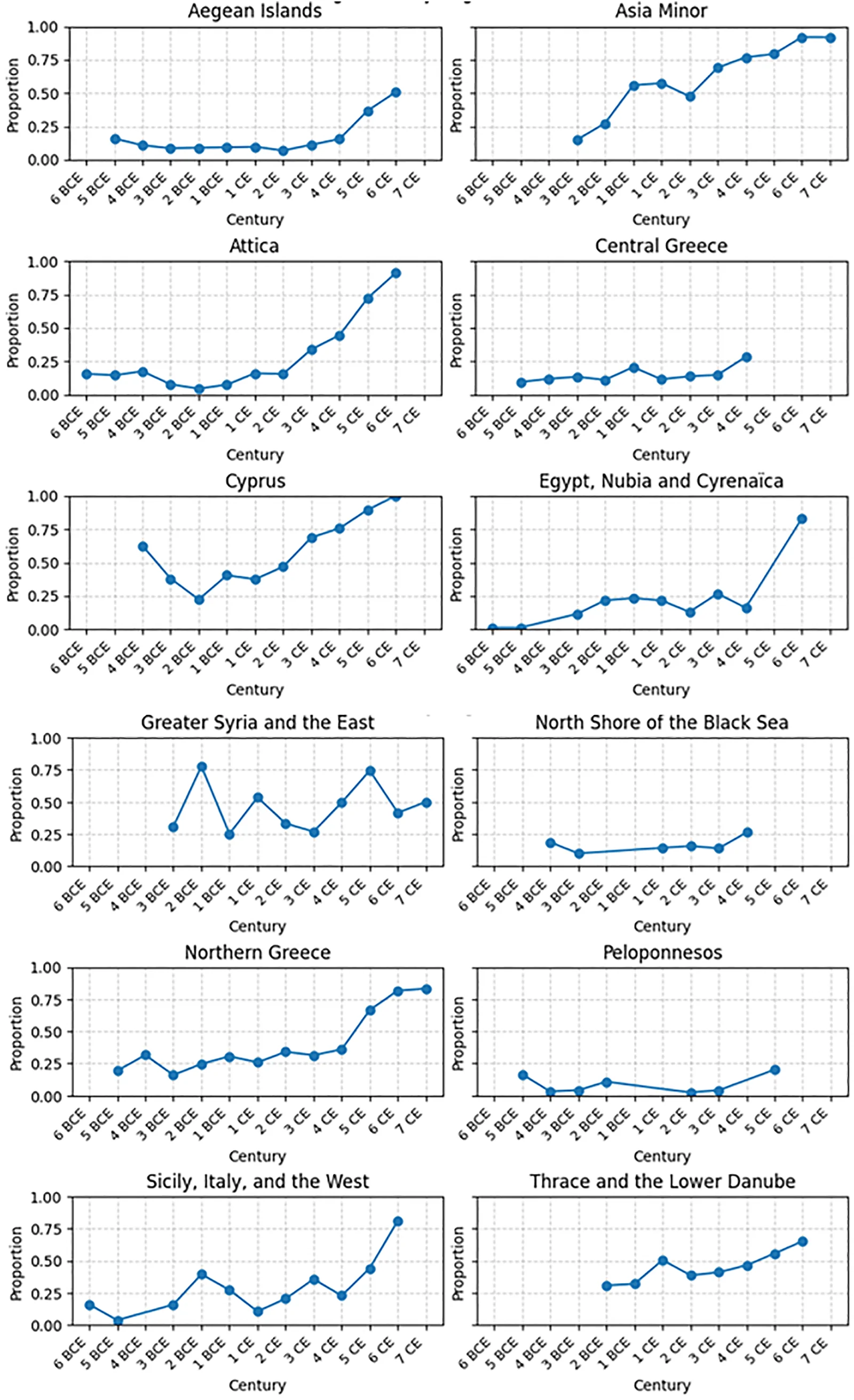
Breakdown of votive proportions. Plots of proportions of votive to all dedications in 12 regions, by century.

This reveals distinct regional profiles:

- Low votive prevalence (pre-fourth century): Attica, Aegean islands, Central Greece, Peloponnese, Black Sea north shore consistently show low proportions (<15–20%), though Attica sees a slight rise in the High Empire.
- High votive prevalence: Asia Minor stands out with consistently high and rising proportions. Syria/East, Cyprus, and Thrace also exhibit higher proportions than the ‘Western’ Greek areas.
- Intermediate or variable: Northern Greece and Egypt fall between these groups. Sicily and Italy show variable patterns based on limited data per century.

This suggests two broad currents in the Greek East: an ‘Eastern’ zone (Asia Minor, Syria, etc.) where explicit vowing in inscriptions was more common earlier and increased more dramatically, and a ‘Western’ zone (mainland Greece, Aegean) where it remained less prevalent until the general rise in Late Antiquity. The overall temporal trend shown in Fig 1 is thus heavily influenced by the patterns in high-inscription regions, particularly Asia Minor. However, almost all regions show a general rise over time and a distinct rising trend in late antiquity.

The investigation of the unusual character of Asia Minor in the corpus requires its own article, with greater geographic resolution and additional of spatial/archaeological context. It is not only an artifact of recovery bias since Asia Minor is not, in general, more investigated than mainland Greece, and we have shown that the percentage of editor restorations leading to identification as votives is relatively low there.

Another phenomenon indicated by the map (Fig 3) is that almost all the large circles are blue. This indicates that dedication inscriptions coming from sites with many inscriptions are usually non-votive, while votive inscriptions appear much more dispersed, and come from smaller sites. Indeed, this phenomenon is clearly shown by dividing the corpus into brackets by number of inscriptions found in a single place (Fig 5).

**Fig 5:**
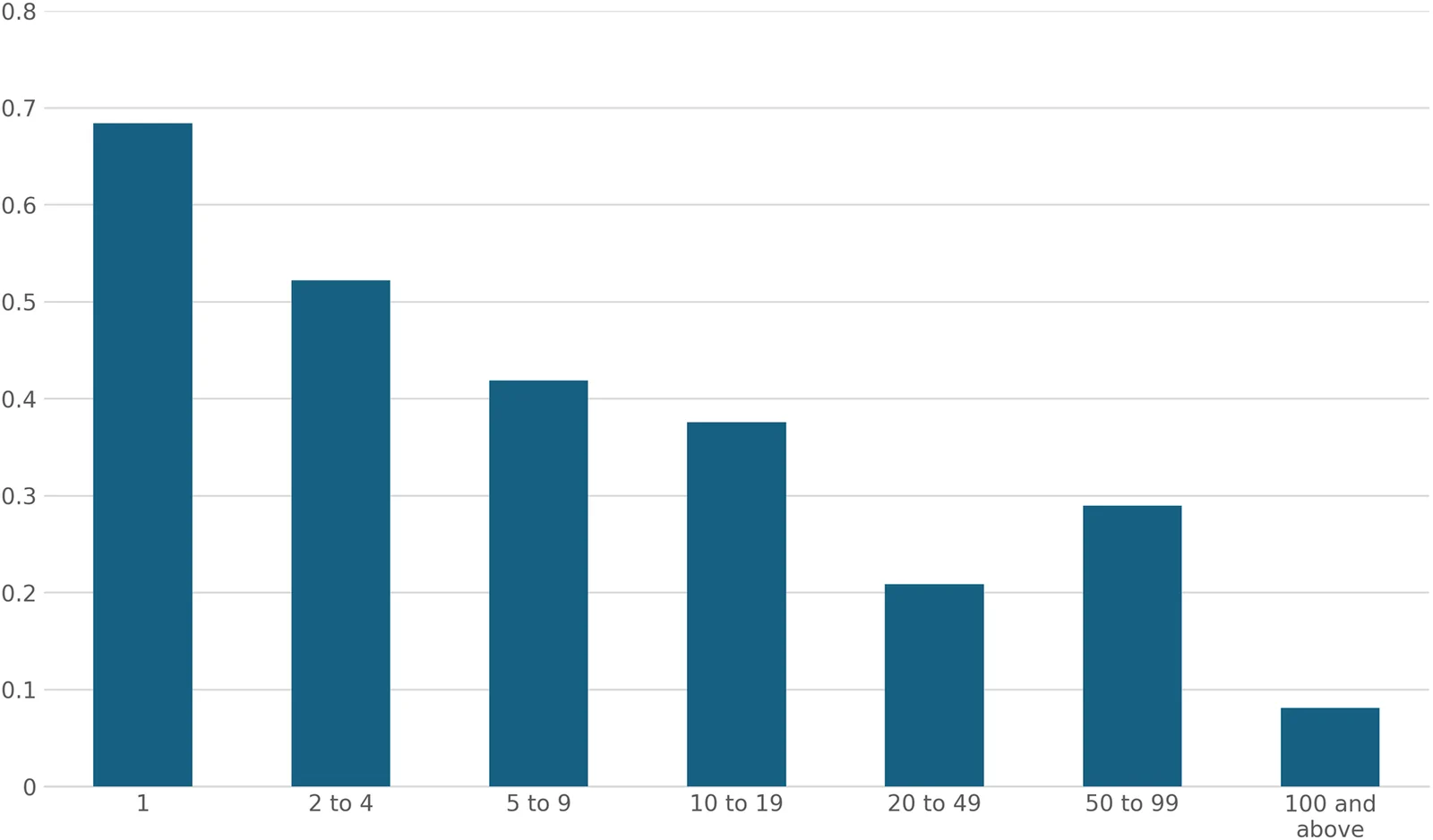
Votives proportion by number of inscriptions. proportion of votives to non-votives in site, according to number of inscriptions found in site.

This analysis shows very clearly that sites with many dedication inscriptions tend to be not-votive. This is especially salient in the larger sites – only one site, the sanctuary of Men Askaenos, includes more than 100 votive dedications (221 inscriptions), compared to 13 sites with more than 100 non-votive dedications (a total of 2772 inscriptions). One option of explaining this phenomenon is that it is an artifact of time and space, that is, that sites with many inscriptions were more common in times and places where votives were less prevalent (e.g., Classical mainland Greece), and vice versa. To check this, I ran a logistic regression controlling for region and chronological period. It showed that log site size remained a significant negative predictor of the presence of votive terminology (β = −0.050, p = 0.009). The corresponding odds ratio (0.95) indicates that inscriptions from sites with larger epigraphic corpora are systematically less likely to contain explicit votive language, even once differences in time and place are taken into account. This finding may tentatively suggest a connection between the experience of the worshipper in smaller, local, sites, which may be more conducive to the closer votive relationship between deity and worshipper than the larger sites. However, a more thorough examination of the evidence is needed to substantiate this idea.

### Deities

Having observed the spatio-temporal trends, we turn to the recipients of these dedications. Similarly, we can ask whether this rise in votive terminology appears across the board for all deities or if it is limited to certain ones. Perhaps, for example, the expansion is a matter of new divinities which are worshipped in this way replacing old ones, rather than a change in the worship of the same deity or sanctuary.

Theoretically, any deity can be the recipient of a vow – this is part of the inherent role of deities in Mediterranean religion. But in practice, certain deities (and epithets of deities) appear as the recipients of vows much more than others. Even before identifying these, the extreme diversity in the votive profile of deities indicates that votive inscriptions (and, by extension, vowing) are not a general marker of religiosity, but are intertwined with specific practices and choices of the worshippers. Table 1, listing deities with ≥10 dedications ordered by the percentage of votive offerings they receive, reveals strong preferences. Deities like Men (83%), Holy and Just (*Hosios kai Dikaios*) (83%), Meter (39%), and those with epithets like Highest (*Hypsistos*) (58%) and Listener (*Epikoos*) (45%) rank highly, while the Emperor (*Autokrator*/*Sebastos*, 1–2%) and traditional Olympians like Athena (6%), Hermes (4%), and Demeter (4%) rank far under the general 20% rate of the whole corpus. So also, to a lesser extent, Egyptian deities – Isis and Serapis (both 12%), and (surprisingly) deities associated with medicine – Apollo (12%) and Asclepius (17%).

**Table 1:** Votives and deities.

Deity	Count all dedications	Count, votives	Percent of votives
Amphiarus	72	0	0%
Agathos Daimon	11	0	0%
Muses	29	0	0%
Rome	10	0	0%
Themis	19	0	0%
Ruler (*Autokrator*)	373	3	1%
Ancestral	211	3	1%
Emperor (*Sebastos*)	300	5	2%
Hestia	83	2	2%
Two Goddesses	30	1	3%
Olympic	321	11	3%
Hermes	178	7	4%
Demeter	274	11	4%
Underworld deities (*Authochtoni*)	23	1	4%
Saviour (*Soter*)	404	21	5%
Pan	19	1	5%
Athena	791	45	6%
Kore	188	13	7%
Gods (*Theoi*)	761	54	7%
Heracles	150	11	7%
Dionysius	171	14	8%
Ammon	24	2	8%
Revealed (*Epiphanes*)	35	3	9%
Aphrodite	323	29	9%
Serapis	361	42	12%
Isis	320	38	12%
Nemesis	42	5	12%
Apollo	794	98	12%
Poseidon	71	9	13%
Goddesses	76	10	13%
Anubis	109	15	14%
Hera	49	7	14%
Hero	131	19	15%
Nymphs	159	24	15%
Hygeia	109	17	16%
Mithras	12	2	17%
Enodia	18	3	17%
Asclepius	494	83	17%
Helios	91	18	20%
Nike	15	3	20%
Artemis	478	100	21%
Telesphoros	13	3	23%
Dioscuri	58	14	24%
Great	318	77	24%
Zeus	987	242	25%
Pluto	12	3	25%
Kurios	70	21	30%
Ancestral Gods	10	3	30%
God (*Theos*)	543	191	35%
Goddess (*Thea*)	182	65	36%
Meter	179	69	39%
Listener (*Epikoos*)	192	86	45%
Highest (*Hypsistos*)	185	108	58%
Children Of Zeus	24	16	67%
Men	298	246	83%
Holy and Just (*Hosios kai Dikaios*)	53	44	83%

Counts and percentages of votives vs. all dedications by deity mentioned

This raises a question: are deities high on the list inherently associated with vowing, or merely popular in regions/periods (like Roman Asia Minor) where vowing was epigraphically common? To disentangle this, I performed a statistical analysis examining the correlation between the presence of votive terms and the presence of each specific deity, controlling for century and region using logistic regression (see details in the material and methods section). Results (Table 2) report deities with statistically significant associations (p < 0.05) in the fully controlled models, presenting odds ratios (ORs), 95% confidence intervals (CIs), and p-values. Full regression outputs for all tested deities are available in S2 Table.

**Table 2:** Controlled odds ratios for deities receiving votives.

Divinity	n_inscriptions	odds_ratio_ctrl	p_value_ctrl	ci_lower_ctrl	ci_upper_ctrl
Children of Zeus	24	50.1476	0	20.4331	123.074
Men	298	6.6458	0	4.7469	9.3044
Holy and Just (*Hosios kai Dikaios*)	53	5.8981	0	2.7753	12.5346
Listener (*Epikoos*)	192	2.011	0	1.4573	2.7751
Artemis	477	1.6477	0.0001	1.2736	2.1316
God	540	1.455	0.0004	1.1808	1.7928
Aphrodite	323	0.5659	0.009	0.3692	0.8674
Nymphs	159	0.5631	0.0246	0.3413	0.9293
Athena	791	0.545	0.0003	0.3921	0.7576
Kore	188	0.4134	0.0033	0.2296	0.7446
Dionysius	171	0.3507	0.0004	0.1966	0.6255
Gods	752	0.3093	0	0.2283	0.4191
Epiphanes	35	0.286	0.0493	0.0821	0.9961
Demeter	274	0.2288	0	0.1232	0.4247
Hermes	178	0.1728	0	0.0777	0.3843
Soter	403	0.1502	0	0.0936	0.2412
Olympic	321	0.141	0	0.0753	0.2641
Hestia	83	0.1205	0.0035	0.0291	0.4994
Ancestral	211	0.0976	0.0001	0.0307	0.3096
Authochtoni	23	0.0723	0.0112	0.0095	0.5503
Sebastos	300	0.0274	0	0.0112	0.0669

Odds ratios, p values, and CIs for controlled models of the correlations of votive terms and deities, p < 0.05.

The controlled analysis largely confirms the patterns seen in Table 1. I cannot comment on each deity, but some general trends are clear, both concerning deities which do receive votives and those who do not. Foremost among the former are deities common in Roman Asia Minor: Men, Meter and the various types of Zeus common in these sanctuaries, as well as epithets emerging especially in the East in the Roman Imperial period: Highest (*Hypsistos*), Holy and Just (*Hosios kai Dikaios*), Great (*Megas*), Lord (*Kurios*) (see discussion in [12,94]). The preponderance of deities of non-Greek origin (e.g., Asia Minor, Egypt, and Syria/Palestine/Israel) in this list is striking.

Deities more associated with the netherworld, on the other hand, such as Hermes or Hecate, were not associated with vowing, nor were some significant Greek deities such as Athena, Dionysus, or Hera, who certainly had a significant number of overall dedications. Significant counter-examples are Artemis and Zeus, with a high percentage of votives. A possible explanation for the prevalence of these deities on the votives list is that they were more commonly joined with local deities in Asia Minor and the East (compared to, e.g., Aphrodite, Athena or Dionysus); and these local deities were traditionally the target for votives. Most pronounced is the rarity of vows addressed to Emperors, especially considering the large number of dedications to them, and their usage in a period when votive terms were overall relatively common.

### Consecration terms

A surprising find is that the rise in votive terminology over time correlates generally with a decline in consecration terminology (the verbs *kathiero* and *anatithemi*, and nouns *anathema* and *dora*), despite a slight rise in both in the Imperial Roman period (Fig 6).

**Fig 6:**
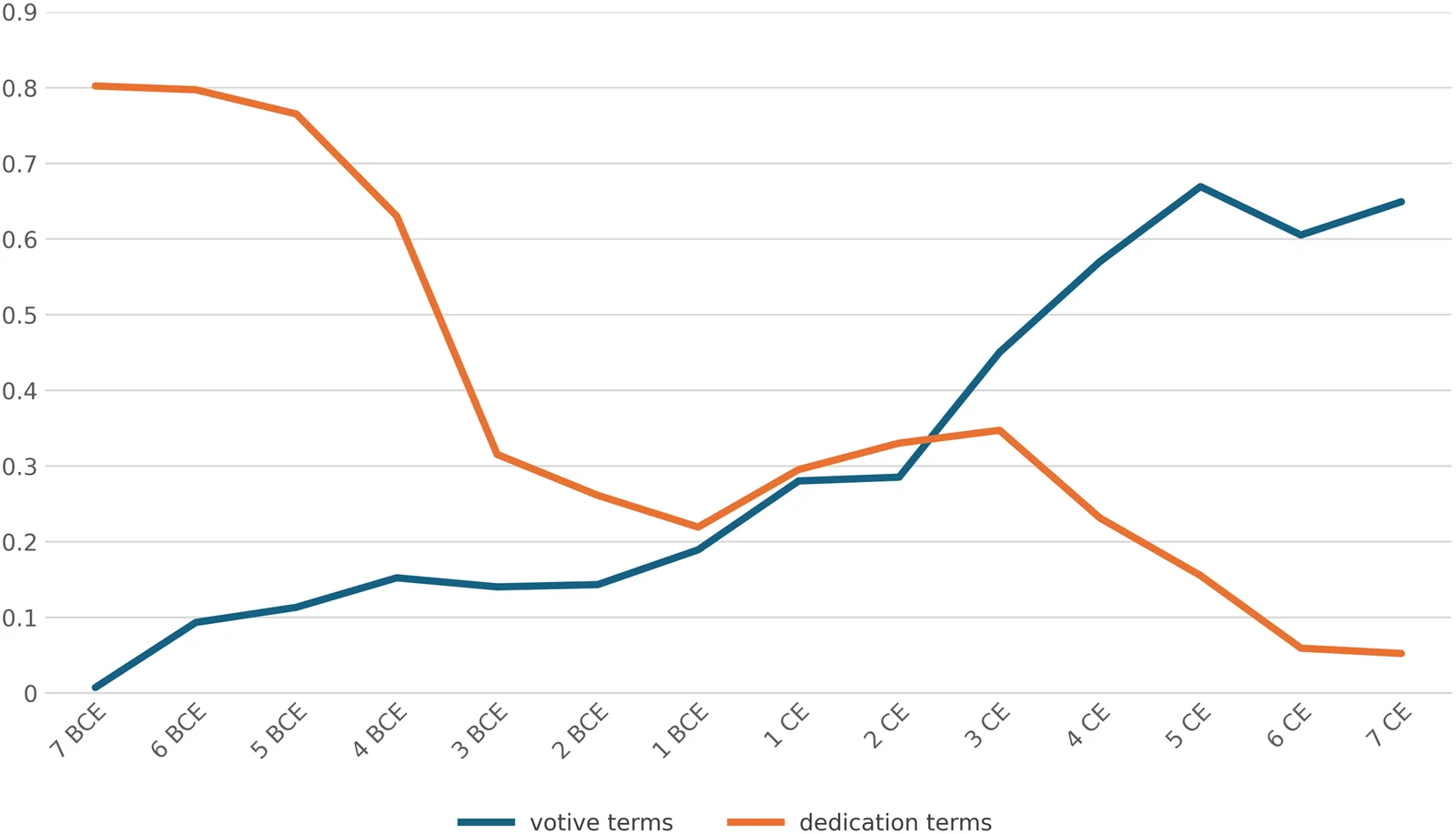
Votive vs. consecration terms. Votive vs. consecration terms as a proportion of all dedication inscriptions.

### Motives

Table no. 3 (Table 3) shows that the mention of additional motives for the dedication in the inscriptions is negatively correlated with votive terminology. For example, “according to a dream” (*kat’onar*) and “according to a commandment” (*kata prostagma*/*keleusin*) very rarely occur together with votive terminology (2% and 7%, respectively). This is not unreasonable, since a vow is itself a motive, giving two motives is superfluous or could even be contradictory.

**Table 3:** Motives and votives.

Motive	all	Votive	Percent
Following a dream	101	2	2%
As firstfruits	165	8	5%
Following a commandment	221	16	7%
As tithe	334	30	9%
Following an oracle	11	1	9%
As thanksgiving	1062	127	12%

Counts and percentages of votives vs. all dedications by motive mentioned

### Reasons

Relative to motives, some reasons – i.e., on behalf of whom or what the dedication was given – are much more associated with votives. I checked for various motives (for friends, for health, for property) appearing in the inscriptions but only those associated with family members and soteria (a broad term, which can be translated as health, well-being, or salvation [95]) appeared in large numbers with votives. In total, family members appeared in 706 dedication inscriptions, 244 of which were votive – 34%; soteria in 81 out of 364 inscriptions – 22%.

However, plotting the proportion of votive inscriptions mentioning family and soteria out of all inscriptions mentioning these reasons, and comparing this with the general proportion of votives out of all dedications for each century (Fig 7), shows this association was highly variable across time. In this graph, the y axis denotes the degree to which a mention of this class of reasons is associated with a greater proportion of votive than non-votive, so that “0” means the same proportion as that of all votives to non-votives. This shows that dedications on behalf of family were almost always much more associated with votives than with non-votives, but this was especially true in the Classical period, with the association declining steadily until the second century BCE, when it started rising again until Late Antiquity. Soteria, on the other hand, start to appear in votive dedications in the third century BCE when they are more associated with votives than with non-votives, but this falls significantly by the first century BCE, and remains at or under the general proportion. Thus, these two reasons show very different chronological votive profiles.

**Fig 7:**
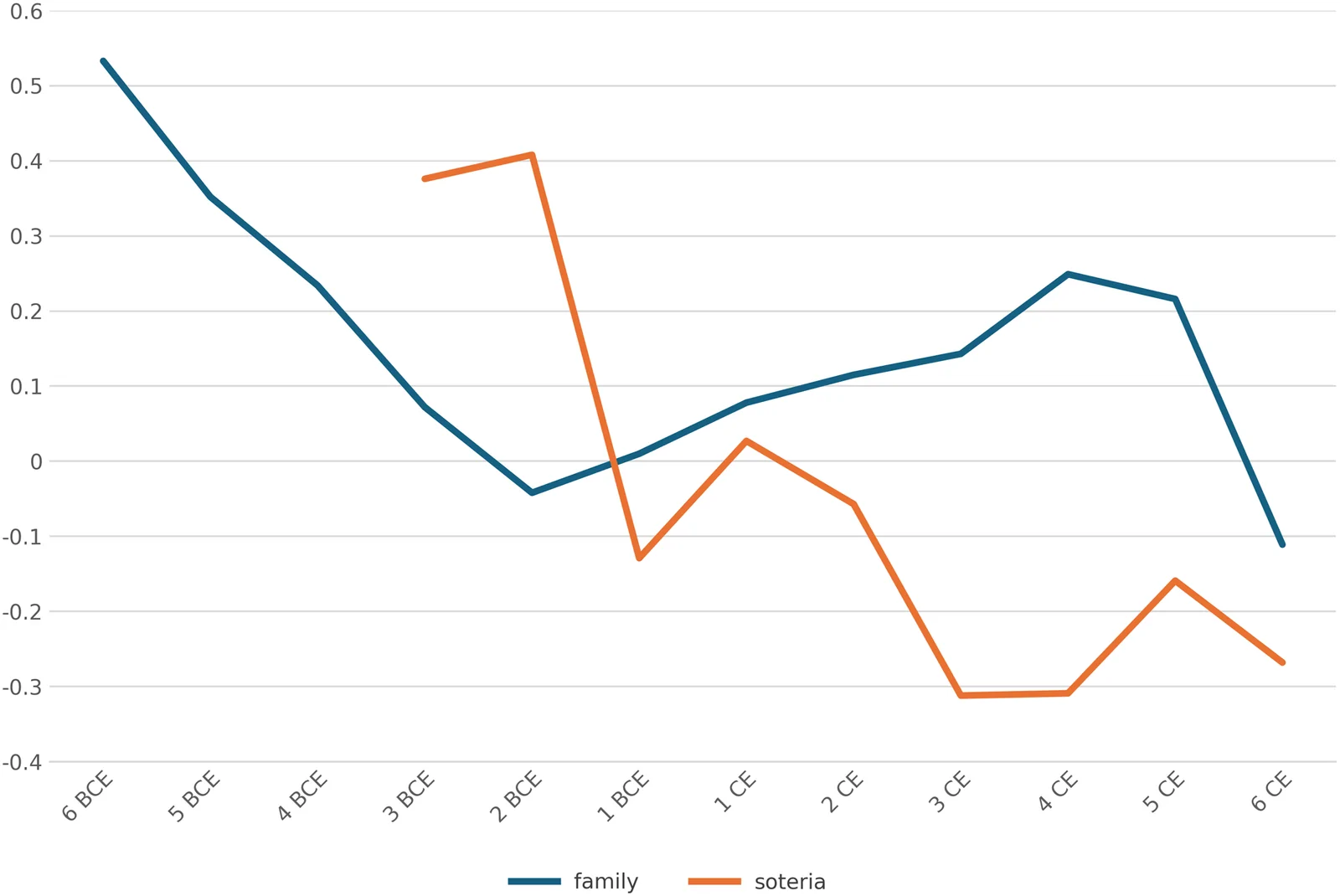
Association of votives with reasons. Proportion of votive dedications on behalf of family or soteria out of all dedications on their behalf, relative to the general proportion of votives to all dedications.

Plotting the percentage of votive inscriptions mentioning family and soteria out of all votive inscriptions over time (Fig 8) shows that vows on behalf of family members are a very stable portion of votive inscriptions, with family members consistently mentioned in between 4% and 7% over the whole period. This contrast somewhat with vows for soteria, which do not appear before the third century BCE, stay at around 1% throughout antiquity, and then rise precipitously in late antiquity, when they become much more popular.

**Fig 8:**
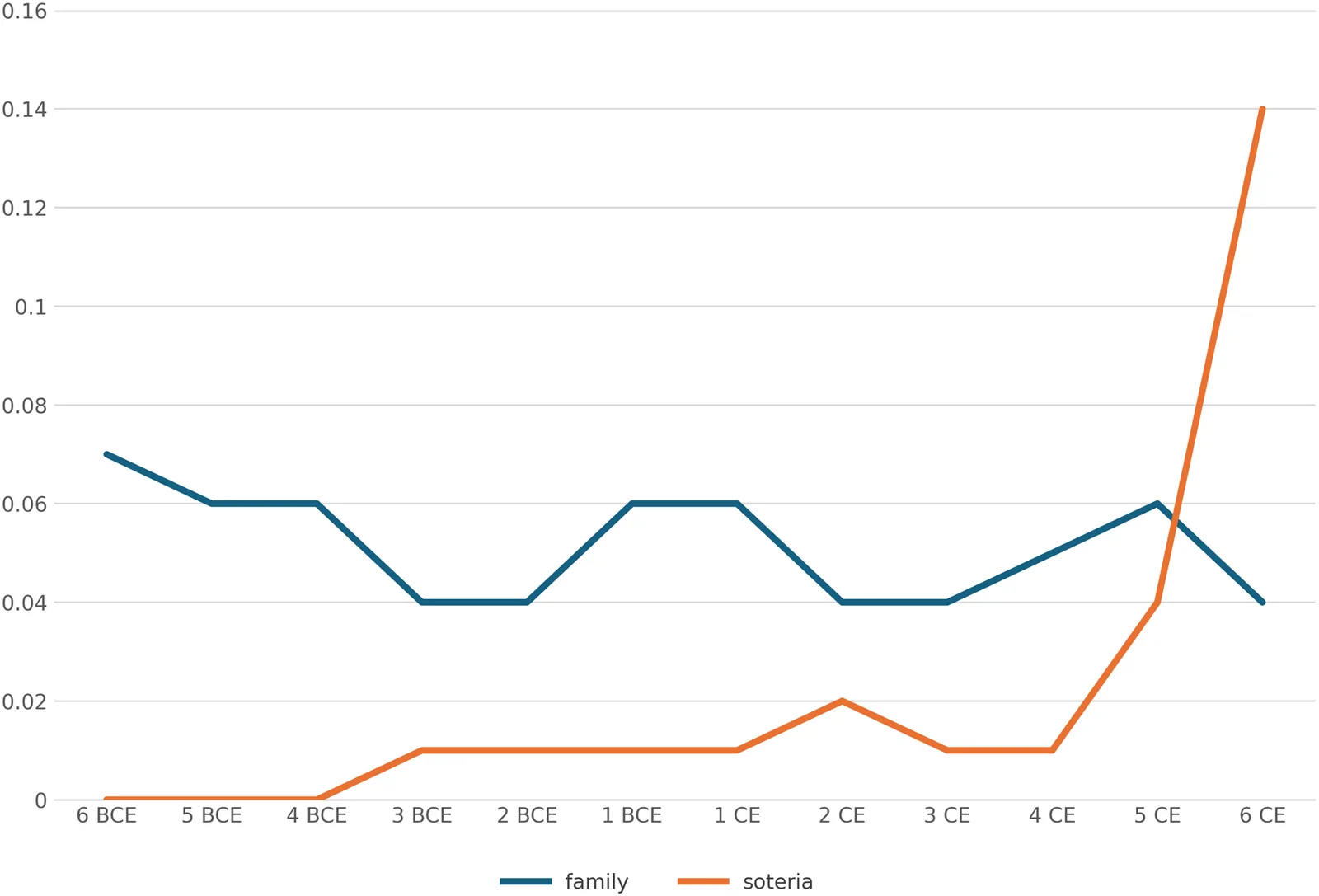
Proportion of votives with reasons. proportion of votive inscriptions mentioning family and soteria out of all votive inscriptions, by century.

### Verb and noun forms

Fig 9 shows the main forms of *euche/ euchomai* in votive inscriptions. As before, the forms show significant change over time but also some continuity. The aorist participle *euchamenos/euchsamenos* (which can be translated as “having vowed”) was most popular in the classical period and declined relatively after this, but its use nevertheless continued also in later periods, well into Byzantine dedications. The accusative noun form, *euchen*, frequently preceded by a preposition such as *kata*, jumped in popularity in the third century BCE and remained so into later antiquity. However after the fifth century CE it declined significantly, to be replaced by the genitive form (*euches*), usually preceded by *hyper*, and popular especially in Christian contexts. This change is well known in scholarship. Some other rarer forms also existed especially towards the end and beginning of the corpus.

**Fig 9:**
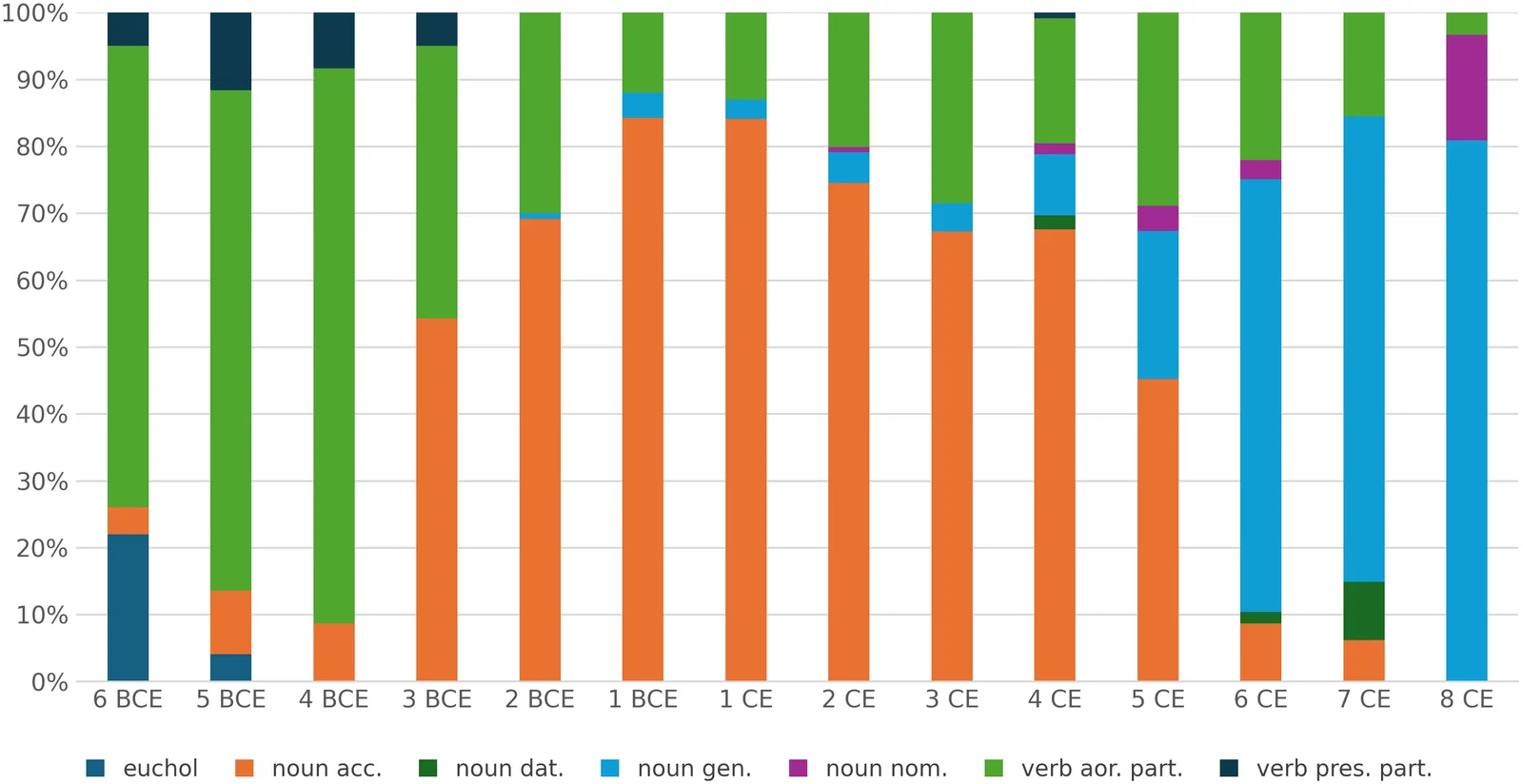
Forms of *euche/ euchomai*, by century.

Though the forms are different there is no clear indication as to a change in meaning – in all cases, the inscription seems to reflect the vower’s decision to fulfil their vows by dedication and to record this fulfilment. More generally, the change in forms seem to reflect conventions of periodization of antiquity (classical, Hellenistic/Roman, and Christian), though interestingly there is no significant change in terminology following the Roman expansion to the East.

I examined more closely the expansion of the two terms which seem to be innovations of a certain period: the use of the accusative *euchen* (frequently preceded by *kata* or *kat’*) in the Hellenistic period, and the term *hyper euches*, “for a vow/prayer”, used usually by Christians, to see if their origins could be determined. This was performed by finding the earliest place and time of inscriptions including these terms, finding the chronological and geographical distance of later inscriptions from the earliest ones, and calculating the correlation between the chronological and geographical distances. However, no clear pattern with statistical significance could be found in geographical expansion, perhaps a result of the lack of precise dating for most inscriptions making it difficult to pinpoint developments over relatively short spans of time.

### Gender

It would be interesting to find if there is an interaction between gender and votive practice, and whether this changes over time. Throughout the Greek world, women frequently inscribed dedications, both votive and non-votive [96,97], in substantial numbers. I analyzed the data to find whether votive dedications were more commonly given by women than men than non-votive dedications (Fig 10); the method of tagging is described in the methods section).

**Fig 10:**
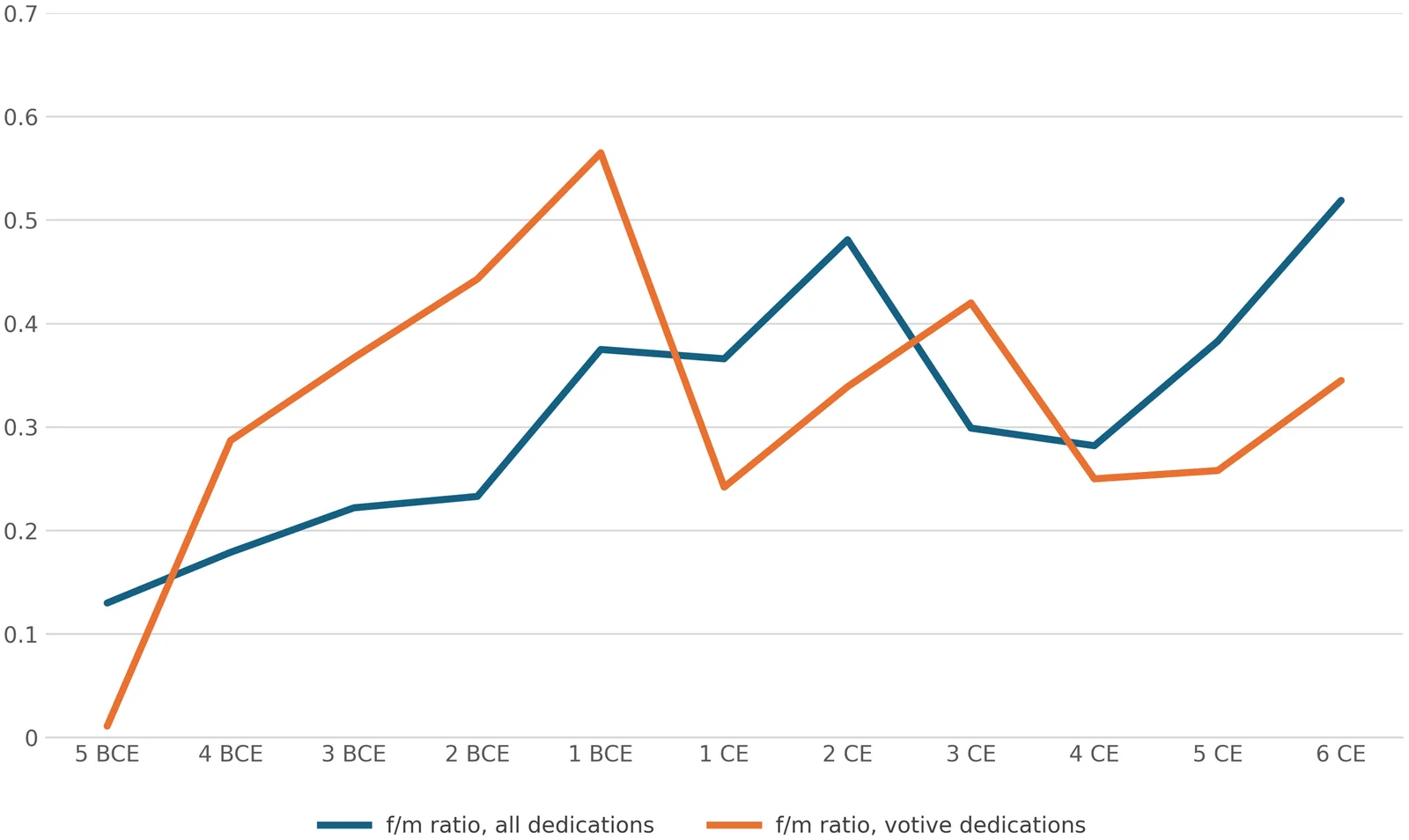
Gender and votives. female to male ratio, votive vs. all dedications.

The results are inconclusive. There is significant variation over time in the representation of women in votive inscriptions. Women are more represented in votive inscriptions than in non-votive inscriptions in the Hellenistic period and the third century CE, but not in earlier or later periods. Further analysis, such as controlling for region or deity, might reveal more nuanced patterns, but based on this initial overview, gender does not appear to be a primary driver of the choice to use votive terminology across the entire corpus.

## Discussion and conclusions

### Key findings

This study employed a distant reading approach to analyze a substantial corpus of approximately 11,000 Greek dedicatory inscriptions and attempt to answer the research questions concerning temporal, geographical and substantive patterns of votive practice. The results suggest that looking at large numbers of inscriptions can indeed reveal trends and correlations that might be less apparent through traditional, localized studies alone. While this quantitative perspective offers valuable insights, it necessarily operates at a level of abstraction and complements, rather than replaces, detailed qualitative analysis.

One key finding is that Greek votive epigraphy exhibits both remarkable long-term continuity and significant changes in specific details over time and space. The fundamental concept of dedicating an object following a vow persisted for over a millennium across the Greek world, despite profound changes in other dimensions of religious culture. However, the frequency of explicitly mentioning the vow (using *euche/euchomai* terminology) increased markedly over time, particularly during the Roman Imperial period and intensifying further in Late Antiquity. A second finding is that the choice of formula by the dedicator was not only individual, as it seems to correlate with many other cultural factors. Rather, it was probably a conscious choice which cohered with the communication strategy with the god chosen by the believer in this specific case, influenced by both society-wide cultural trends and by the choices made by each individual.

The patterns of votive practice in time and space are intertwined. Early Greek dedications in the Classical period included significantly less votives than later ones, and were of course concentrated only in areas where Greek was used. However, even after the expansion of Greek culture eastwards, the core Greek areas remained at a relatively low levels of votives, with the higher levels seen especially in Asia Minor and environs, though also in northern Greece. It is significant that this rising trend in votive dedications was found also for Latin by Ehmig [38], who showed that Republican inscriptions were characterized by *donum* (gift) dedications, Imperial ones by *votum* (vow) dedications. This general pattern shows that even though vowing was always a possible religious strategy in the Eastern Mediterranean, it was more suitable for the religious proclivities of Eastern (originally non-Greek) rather than Western (originally Greek) societies.

### Distant reading: insights and challenges

This work underscores the indispensable role of open, accessible, and well-structured digital databases like GIST/I.PHI for enabling large-scale epigraphic research. The ability to query and analyze thousands of texts with associated metadata opens up new avenues for historical inquiry. However, the study also highlights some challenges. While automated tagging and analysis are powerful, significant improvements in accuracy and nuance would likely require more extensive manual cleaning, verification, and tagging of the data, particularly concerning complex categories like motives or the precise nature of the dedicated object. The analysis also confirmed the critical importance of geographical and chronological slicing of the data; aggregated results can mask significant regional and temporal variations.

This article attempts a distant reading approach to ancient dedicatory epigraphy. Some researchers have highlighted the dangers of this approach: using inscriptions as data takes them out of context, and turns them from singular artifacts, tied to a specific event, into part of a data series. Doing so, it homogenizes them and extracts from them only the textual information that is easily abstractable and digitizable. Tagging inscriptions produces further abstraction as it groups together different lexemes under one tag.

However, it may be argued that any historical endeavor which attempts to produce an analysis over a long period or large area necessarily selects and abstracts significantly from the data; digital analysis exposes such abstractions and allows us to be more aware of them [57]. Furthermore, it is argued that distant reading surfaces differences and temporal trends which are otherwise impossible to spot in close readings. Specifically, for dedicatory epigraphy, while every inscription is unique, the inscriptions are also very formulaic. Worshippers and stone cutters imitated each other and formed an epigraphic culture. The comparative, distant reading approach to some extent reflects this reality in attempting to chart multiple rather than single inscriptions. The possible word choices of the inscription are seen as a repertoire from which worshippers could have chosen, but with the important emphasis that these choices were not all equal but were influenced by geographical, cultural and temporal trends. Thus, the choice to inscribe “dedicated, having vowed” on a dedication would have a different meaning in Roman Asia Minor (where it was all too common) compared to Classical Greece (where it was quite irregular). Reading the corpus as a whole also helps to address a limitation of studies based mainly on close readings. These tend to focus on inscriptions that are longer, more explicit, or narratively richer, because such texts appear more informative.

The analysis further shows that dedicatory inscriptions did not usually combine several different explanations for a dedication. Votive terminology appears only rarely together with references to dreams, divine commands, or other motives. This suggests that worshippers typically selected one main way of framing their dedication, rather than combining different justifications. The choice of formula was therefore not random or only a personal choice, but shaped by what was considered appropriate in a given place, period, and cultic setting.

The broader geographical scope of the analysis also complicates assumptions about what counts as “Greek” votive practice, considering the significant divergence between different areas. The widespread use of Greek votive terminology in areas where Greek was only one of several languages used suggests that Greek inscriptions there may reflect local religious traditions as much as inherited Greek ones. Distant reading thus makes it possible to see Greek votive epigraphy not simply as the diffusion of a single tradition from the center, but as a set of practices shaped by regional interaction.

The differentiation in the geographic pattern can be seen also in the clear negative correlation between the number of dedications found in a site and the proportion of votives. It is possible that sites with many inscriptions were simply more prevalent in areas and periods in which votives were less prominent (e.g., sixth-third centuries BCE Attica or the Aegean, which included several large Pan-Hellenic sanctuaries), without any real connection between the number of dedications and the type of ritual practice. Even if this is true, it is striking that inscribed vows are, with some exceptions, a feature of small, dispersed sanctuaries, and more frequently outside of the core Greek areas, rather than large centralized ones in the core Greek area to the shores of the Aegean. Perhaps the type of religiosity of a long-term connection with a specific god, implied in a vow and its fulfilment, was better suited for the cultures of these periods and areas. Thus, we may propose that the importance of vows in Asia Minor appears to reflect an actual difference in its religious character, which can be seen also in the well-known, and unusual, “confession inscriptions” from the same area and period. These inscriptions show that in some areas of Asia Minor (Lydia and Phrygia) epigraphy was considered a medium for narrating and managing one’s relationship with the gods to a greater extent than in other areas [98,99].

It appears to be strongly associated with specific deities, particularly those prominent in Roman Asia Minor (like Men, Meter, and certain epithets of Zeus) and some (though by no means all) of those originating outside the traditional Greek heartlands. Some deities and epithets new to the Roman period – *Hypsistos* and *Hosios kai Dikaios* – clearly stand out in their association with votives. Conversely, the Emperor and certain traditional Olympian figures received comparatively fewer explicit votive offerings. The phenomenon of lack of votives to the emperor was already noted by previous scholars who saw this as an indication of their distinct, perhaps less reciprocal, perceived divine status [100,101]. The low rate of votive dedications for Isis, Apollo and even Asclepius, deities commonly associated with healing, is surprising considering the common association in scholarship of vowing with crisis situations and specifically with medicine [21–23]. Whatever the reason, certain details of dedicatory ritual were clearly considered as much more appropriate when turning to certain gods rather than others, even if the general practice was similar. This preference goes beyond the background regional and chronological variation. It may have been related to the perceived personality and abilities of the relevant deity, or to the social and cultural characteristics of the specific communities worshipping these deities. The highly significant difference in the proportion of votive inscriptions between different deities supports the view that votive terminology is not just a decision to apply a fashionable formula, but a decision reflecting a specific religious attitude, appropriate to certain deities more than to others. The importance of selecting the best deities as object for sacrifice, prayer or vow may be seen, for example, in the oracle tablets from Dodona, in which worshippers frequently asked Zeus to which gods they should direct their *euche* (prayer or vow) in order to manage a certain situation [102].

### Religious language, practice and mentality

It is difficult or even impossible, in most cases, to know whether votive terminology indeed reflected a vow rather than a dedication not preceded by a vow, though many scholars take such epigraphic statements at face value. Tri- and bi-lingual inscriptions, in which only one language (typically Latin) mentions a vow while others do not alert us to the possibility that votive terms could become formulaic, and become simply synonymous with dedication [51]. Thus, people may have performed vows without inscribing them (either at all or with an inscription not containing votive terminology), or vice-versa. However, the very use of such terms must have had some meaning for the writers and readers of these texts. Votive terminology reflects a relationship with the deity over time, in which the dedicator at the moment of dedication completes an obligation created towards the deity in the past, sometimes the far past. Thus, votive terminology does not necessarily mean that the dedicator feels closer to the god than when not using such terminology, but it does indicate a protracted relationship, or, sometimes, one created in a moment of stress requiring special assistance, as opposed to a decision made close to the time of dedication: “Every dedication set up in fulfilment of a vow (εὐχήν, κατ᾿ εὐχήν) presupposes a prayer addressed to a… god or goddess that a man or woman considered heard and fulfilled” [103]. The results of this analysis can show us in what times, in what places and (to a limited degree) under which circumstances people decided to engage in such relationships with the divine.

The argument that there is a specific mindset associated with vowing is bolstered by the finding of a negative correlation between consecration and votive terms. One option is that there is limited space (physical and mental) for writing on a dedication, and that the rise of one type of terminology obviates the other. Since it is obvious that a vowed object was also consecrated, consecration terms might have been deemed less needed. Alternatively, the change can be seen as more significant: a rising emphasis on the long-term connection between the worshipper and the deity implicit in the vow rather than on the specific action of consecration. This would cohere with some scholarship of the past few decades, which posits a shift towards more personal and relational forms of piety in the Roman period, more individual commitment to specific gods, and more extensive and emotional language in inscriptions from diverse members of society [104–107]. However, the two phenomena may be unrelated: in the pre-Roman period votive terminology is relatively steady, and the decline in consecration terminology may have independent causes. In the Roman period both seem to rise in tandem, probably because dedication inscriptions become generally more detailed and provide more information. After the fourth century, votive terminology became standard in Christian contexts while consecration terminology fell out of use.

Paul Veyne has argued that in the Roman imperial period, oracles became a more prevalent form of communication between gods and private people, as part of a general shift in religious mentality towards more authoritative relationships between gods and humans [39]. This shift, he argued, extended also to vows which were sometimes ordered by an oracle rather than given freely [39]. However, the analysis of the votive corpus shows that vows as a response to a divine dream or a command were very rare compared to divine commands resulting in non-votive dedications. Presumably, other motives like thanksgiving or divine command were not needed in votives because the vow itself served as the primary justification. The brief examination of people or aspects on behalf of which vows were performed shows that families were frequently a focus of the vow, more so than non-votive dedications. It would appear that the needs of one’s family were more amenable to the type of communication with the gods which vows offered. However, this connection was not at all stable over time, and I find it difficult to explain why the changes occurred. Dedications on the behalf of well-being or salvation, on the other hand, are less associated with vowing, though again, it is difficult to say why exactly this would be so.

The dynamics of the various dimensions of vowing indicate that the change from pre-Christian to Christian ritual was not abrupt. While some formulae changed, others remained similar. Though there is a general rise in the Late Antiquity in the rate of votive compared to non-votive dedication, this continued in many regions on earlier trends. Though the study did not investigate Christian inscriptions (e.g., coming from churches) separately from non-Christian ones, it is clear that most inscriptions in the fifth and sixth centuries CE were Christian, and that while the formulae generally changes to *hyper euches*, other dimensions of the votive inscriptions examined here – such as motives and gender – do not change decisively. Though this is not surprising, it is yet another example for how many local religious temple practices were translated fluidly into Christian idiom.

### Further research

This investigation is preliminary. The base corpus, though very significant, is not up to date, and requires further cleaning and disambiguation of double attestations, and can also be improved, for example, by better dating of the inscriptions in certain cases. Also important is the contextual information of the inscriptions, such as artwork associated with them or their findspots: it clearly matters whether an inscription was found in a church, a synagogue or a temple, but the current study could not relate to this. This will facilitate understanding whether an inscription came from Christian, Jewish or other contexts, and to analyze the corpus accordingly. Similarly, dedication inscriptions may also be influenced by the media on which they are written (e.g., stone, mosaic, metal). Other dimensions such as the status of the dedicator or the object dedicated can also be investigated. Furthermore, there is a need for more precise geographical research of the subject, by using smaller geographical units in the analysis. From the results of this study, it is clear that a separate study of Asia Minor may yield interesting, and more precise, results.

## Supporting information

S1 Table Keywords, regexes and tags. (CSV)

S2 Table Full regression outputs for all tested deities. (CSV)
